# Clinical and genetic investigation of ichthyosis in familial and sporadic cases in south of Tunisia: genotype–phenotype correlation

**DOI:** 10.1186/s12920-021-01154-z

**Published:** 2022-01-05

**Authors:** Mariem Ennouri, Andreas D. Zimmer, Emna Bahloul, Rim Chaabouni, Slaheddine Marrakchi, Hamida Turki, Faiza Fakhfakh, Noura Bougacha-Elleuch, Judith Fischer

**Affiliations:** 1 Laboratory of Molecular and Functional Genetics, Faculty of Sciences of Sfax, Sfax University, Sfax University, Street of Soukra km 4, BP 1171-3000, Sfax, Tunisia; 2 Faculty of Medicine, Institute of Human Genetics, Medical Center, University of Freiburg, Freiburg, Germany; 3 Department of Dermatology, CHU Hedi Chaker, Sfax University, Sfax, Tunisia

**Keywords:** Ichthyosis, *TGM1*, *NIPAL4*, *CYP4F22*, *CERS3*, *ABCA12*

## Abstract

**Background:**

Ichthyosis is a heterogeneous group of Mendelian cornification disorders that includes syndromic and non-syndromic forms. Autosomal Recessive Congenital Ichthyosis (ARCI) and Ichthyosis Linearis Circumflexa (ILC) belong to non-syndromic forms. Syndromic ichthyosis is rather a large group of heterogeneous diseases. Overlapping phenotypes and genotypes between these disorders is a major characteristic. Therefore, determining the specific genetic background for each form would be necessary.

**Methods:**

A total of 11 Tunisian patients with non-syndromic (8 with ARCI and 2 with ILC) and autosomal syndromic ichthyosis (1 patient) were screened by a custom Agilent HaloPlex multi-gene panel and the segregation of causative mutations were analyzed in available family members.

**Results:**

Clinical and molecular characterization, leading to genotype–phenotype correlation in 11 Tunisian patients was carried out. Overall, we identified 8 mutations in 5 genes. Thus, in patients with ARCI, we identified a novel (c.118T > C in *NIPAL4*) and 4 already reported mutations (c.534A > C in *NIPAL4*; c.788G > A and c.1042C > T in *TGM1* and c.844C > T in *CYP4F22*). Yellowish severe keratoderma was found to be associated with *NIPAL4* variations and brachydactyly to *TGM1* mutations. Two novel variations (c.5898G > C and c.2855A > G in *ABCA12*) seemed to be features of ILC. Delexon13 in *CERS3* was reported in a patient with syndromic ichthyosis.

**Conclusions:**

Our study further extends the spectrum of mutations involved in ichthyosis as well as clinical features that could help directing genetic investigation.

## Introduction

Ichthyosis is a heterogeneous group of Mendelian cornification disorders that includes syndromic and non-syndromic forms. Autosomal Recessive Congenital Ichthyosis (ARCI) and Ichthyosis Linearis Circumflexa (ILC) belong to non-syndromic forms. ARCI is characterized by abnormal desquamation over the whole body due to a dysfunctional skin permeability barrier and altered lipid composition. It is a rare skin disease affecting around 1 in 200,000 individuals [1, 2].

ARCI defines three major clinical subtypes with varying severity degrees, including the spectrum of Congenital Ichthyosiform Erythroderma (CIE), Lamellar Ichthyosis (LI) and Harlequin Ichthyosis (HI). HI is the most severe form of inherited ichthyosis and can be fatal. The clinical features of this subgroup include thick, plate-like scales with severe ectropion, flattening of the ears and eclabium [3, 4]. LI form is a milder phenotype, characterized by a thick dark/brown scale covering the majority of the body and palmoplantar keratoderma [5]. LI patients are usually born as collodion babies. CIE phenotype is rather characterized by fine, white scales and erythroderma. Like LI, CIE babies are born as collodion. ILC is a rare form of ichthyosis characterized by polycyclic patches bordered by a double-edged scale.

Beside clinical heterogeneity, a large genetic heterogeneity was described in ichthyosis. Thirteen genes, encoding proteins essential for skin barrier formation [6, 7] have been involved: *TGM1* (MIM:190195) [7], *NIPAL4* (MIM:609383) [8], *ALOXE3* (MIM: 607206) [9], *ABCA12* (MIM: 607800) [10], *SDR9C7* (MIM: 609769) [11], *PNPLA1* (MIM: 612121) [12], *SULT2B1* (MIM: 604125) [13], *CYP4F22* (MIM: 611495) [14], *CERS3* (MIM: 615276) [15], *ALOX12B* (MIM: *603741)* [16]*, LIPN (MIM: 613924)* [17]*, SLC27A4*(MIM: 604194) [18], and *ST14* (MIM: 606797) [19].

A major characteristic of ARCI is partially overlapping phenotypes and genotypes between LI and CIE [20] (Table 1). Therefore, it would be difficult to determine the specific genetic background for each form. However, involvement of particular genes in specific forms was proposed.

**Table 1:** Summary table of clinical signs and genetic background associated with ichthyosis forms (LI, CIE and ILC) [3]

Characteristics	Ichthyosis form		
	LI	CIE	ILC
Mode of inheritance	AR	AR	AD
Onset	At birth	At birth	At birth or neonatal
Initial clinical presentation	Collodion membrane with ectropion and eclabium; less frequently CIE	CIE or less frequently mild collodion membrane	CIE
Distribution of scaling	Generalized; focally pronounced scaling possible	Generalized; focally pronounced scaling possible	Localized serpiginous, migratory
Scaling type	Coarse and large (plate like)	Fine	Fine
Scaling color	Brownish or dark	White or gray	White
Erythema	Variable, less pronounced	Variable, often pronounced	Variable
Palmoplantar involvement	*CYP4F22*: pronounced lichenification and mild keratoderma	*NIPAL4*: pronounced keratoderma	No
	*TGM1*: frequent palmoplantar involvement	*TGM1*: frequent palmoplantar involvement	
Scalp abnormalities	Scarring alopecia possible (often with *TGM1*)	Scarring alopecia possible	No

*LI* lamellar ichthyosis; *CIE* congenital ichthyosiform erythoderma; *ILC* ichthyosis linearis circumflexa; *AR* autosmic recessive; *AD* autosomic dominant

The current study focused on 11 newly recruited Tunisian patients affected with either non-syndromic ichthyosis forms (8 with ARCI: 4 with CIE and 4 with LI; 2 with ILC) or autosomal syndromic ichthyosis (1 patient). We tried to highlight some clinical features related to specific genes that could facilitate genetic diagnosis. We reported three novel missense mutations in two different consanguineous families, as well as 5 known disease-causing variants in 4 different genes in the remaining subjects. In silico analysis was performed in order to predict the effect of novel mutations on each corresponding protein level.

## Methods

1. Patients

A total of 11 patients with different ichthyosis phenotypes were enrolled. Among them, five belonged to two consanguineous families: A (A3, A4 and A7) and F (F1 and F2). The other six cases (M1, B1, C1, Y1, K1 and E1) were sporadic. Ten out of 11 patients were affected with non-syndromic ichthyosis (8 with ARCI: 4 with CIE and 4 with LI; 2 with ILC). Only one patient suffered from autosomal syndromic ichthyosis.

The study protocol conforms to the approval of the local ethical committee of CHU Hedi Chaker, Sfax, in compliance with the Declaration of Helsinki. Written informed consent was obtained from all study participants or the parents for minor children.

2. DNA extraction

Total genomic DNA was extracted from patients and available members’ family using the phenol–chloroform standard procedure [21]. The DNA concentration was measured with a Qubit 2.0 fluorometer (Life Technologies, Carlsbad, CA, USA).

3. Next generation sequencing

Next generation sequencing (NGS) was performed using a custom Agilent HaloPlex multi-gene panel with 32 ichthyosis linked genes comprising a target size of ~ 64 kbp. The average sequencing depth was 420.3x. A rate of 97.6% of the target position had at least 20 × in depth. Sequencing was performed on an Illumina MiSeq sequencer using MiSeq reagent kit v2 (2 × 150 bp). Reads were aligned using bwa (v. 0.7.17) [22] against human hg19, and after recalibration, realignments, and genotype calling with gatk (gatk38 nightly-2017–12-06–1) [23], freebayes (v1.2.0-dirty) [24], vardict (v20180621) [25], bcftools (v.1.9) [26]. Variants were annotated using ANNOVAR [27], Variant Effect Predictor [28] and custom scripts.

4. Sanger sequencing

PCR amplification was performed using a thermal cycler GeneAmp PCR System 2720 (Applied Biosystems, Foster City, CA), in a final volume of 15 μl using 100 ng DNA, 10 μM of each primer, 2 mM dNTP, 10 × PCR buffer, 1U of Taq DNA polymerase (Qiagen) and Q-solution. Primers were generated with primer3 and corresponding sequences are mentioned in Table 2.

**Table 2:** Primers’ sequences used for genes amplification

*Gene*/exon	Primers sequences	
	Forward	Reverse
*NIPAL4*/exon1	5’CATCTAGGTCCCCTGTACTC3’	5’CCTGGGAGGAGAGGATGC3’
*ABCA12*/exon18	5’GGCTGTGCTGTCTAATCCTAGC3’	5’TGTATCTGTAGGAAGCACATTCA3’
*ABCA12*/exon37	5’GTGCGGGCTGAGAGATTAAG3’	5’GGTGTGAGCCACTGTACCTG3’

PCR products were sequenced using Applied biosystem (HITACHI 3500XL analyzer). Sequences were analyzed, using Bio Edit Program and compared with wild type sequences: *NIPAL4* (ENST00000311946.7) and *ABCA12* (ENSG00000141527.18) by BLAST online software.

5. Bioinformatic predictions

Protein sequence alignment across species was performed using CLUSTALW and PolyPhen2 software. The potential functional impact of p.(M63T) mutation on NIPA4 and ABCA12 proteins was predicted using PolyPhen2, SIFT, Mutation Taster, Panther and CADD software.

## Results

### Clinical analysis

The current study included 11 ichthyosis patients with 3 phenotypes. Among them, five belonged to two consanguineous families: A (A3, A4 and A7) and F (F1 and F2). The other six cases (C1, M1, B1, Y1, K1 and E1) were sporadic (Table 3).

**Table 3:** Clinical features, affected genes and characteristics of new genetic variations identified for studied patients with ichthyosis

	ID	A3	A4	A7	F1	F2	C1	M1	B1	Y1	K1	E1
Phenotype	Sex	M	F	M	F	F	M	M	F	M	M	F
	Age at diag*	3	1	6	35	4	34	74	1	19	14	15
	Collodion baby	P	P	P	A	A	ND	P	P	P	P	A
	Plate like scales	P	A	P	A	A	A	A	P	P	P	A
	Fine scales	A	P	A	P	P	P	P	A	A	A	P
	Scale color	Brown	White	Black	Brown	Brown	White	White	Brown	Black	Brown	White
	Erythema	P	P	A	P	A	P	P	A	P	A	P
	Ectropion	P	P	A	A	A	A	P	P	P	A	A
	Alopecia	A	A	A	A	A	A	P	A	A	A	A
	Palmo hyper	P	A	P	A	A	Severe	Severe	P	P	P	P
	Pseudo Ainhum	A	A	A	A	A	P	A	A	P	A	A
	Brachydactyly	A	A	A	A	A	A	P	P	P	A	P
	Ear Deformity	A	A	A	A	A	A	A	P	P	A	A
	Folds Involvement	P	A	P	A	Inguial	A	P	P	P	A	P
	Diagnosis	CIE	CIE	LI	ILC	ILC	CIE	CIE	LI	LI	LI	CIE^•^
Genotype	Affected Gene	*NIPAL4*	*NIPAL4*	*NIPAL4*	*ABCA12*	*ABCA12*	*NIPAL4*	*TGM1*	*TGM1*	*TGM1*	*CYP4F22*	*CERS3*
	Mutation	c.118C > T^N^	c.118C > T^N^	c.118C > T^N^	c.2855A > G^N^ c.5898G > C^N^	c.2855A > G^N^ c.5898G > C^N^	c.534A > C^R^	c.788G > A^R^	c.788G > A^R^	c.1042C > T^R^	c.844C > T^R^	c.(999 + 1_1000-1)_(*1_?)del
	State	H	H	H	H/H	H/H	H	H	H	H	H	H
	State in literature [Ref]	–	–	–	–	–	H [46, 49]	H [42, 51] C.het [52]	H [42, 51] C.het [52]	C.het [51, 53]	C.het [30] H [29]	H [44]
	Exon	1	1	1	18; 37	18;37	4	5	5	7	8	13
Bioinformatic prediction (score)	Polyphen2	0.808^(+)^	0.808 ^(+)^	0.808^(+)^	0.899^(++)^	0.899^(++)^	0.004^(Φ)^	–	–	–	0.955^(+)^	–
	SIFT	0.036^(#)^	0.036^(#)^	0.036^(#)^	0; 0.008^(##)^	0; 0.008^(##)^	0.03^(#)^	–	–	–	0^(##)^	–
	Mutation Taster	0.995^(ψ)^	0.995^(ψ)^	0.995^(ψ)^	1^(ψ)^;0.999^(ψψ)^	1^(ψ)^;0.999^(ψψ)^	0.999^(ψψ)^	1^(ψψ)^	1^(ψψ)^	1^(ψψ)^	0.999^(ψψ)^	–
	Panther	1629^(++)^	1629^(++)^	1629^(++)^	1238^(++)^ 1237^(++)^	1238^(++)^ 1237^(++)^	1629^(++)^	–	–	–	1038^(++)^	–
	CADD	23.5	23.5	23.5	28.4; 32	28.4; 32	28.5	–	–	–	23.9	–
	MAF (GnomAd)	A	A	A	A	A	A	0.00002784	0.00002784	0.000007086	0.00001194	A

*F* Female; *M* Male; * Age at diagnosis (years); *ND* not determined; *Palmo hyper* Palmoplantar hyperkeratosis; *P* Present; *A* Absent; *LI* lamellar ichthyosis; *CIE* congenital ichthyosiform erythoderma; *ILC* ichthyosis linearis circumflexa; *CIE*^•^CIE with ocular defect, *Del* deletion; – does not exist; *H* homozygous; *C.het* composite heterozygous; ^(+)^ possibly damaging; ^(++)^ probably damaging; ^(#)^ damaging; ^(##)^ deleterious; ^(ψ)^: polymorphism; ^(ψψ)^disease causing; ^(Φ)^ benign, *N* new variant; *R* reported variant; *MAF* minor allele frequency

**Fig. 1:**
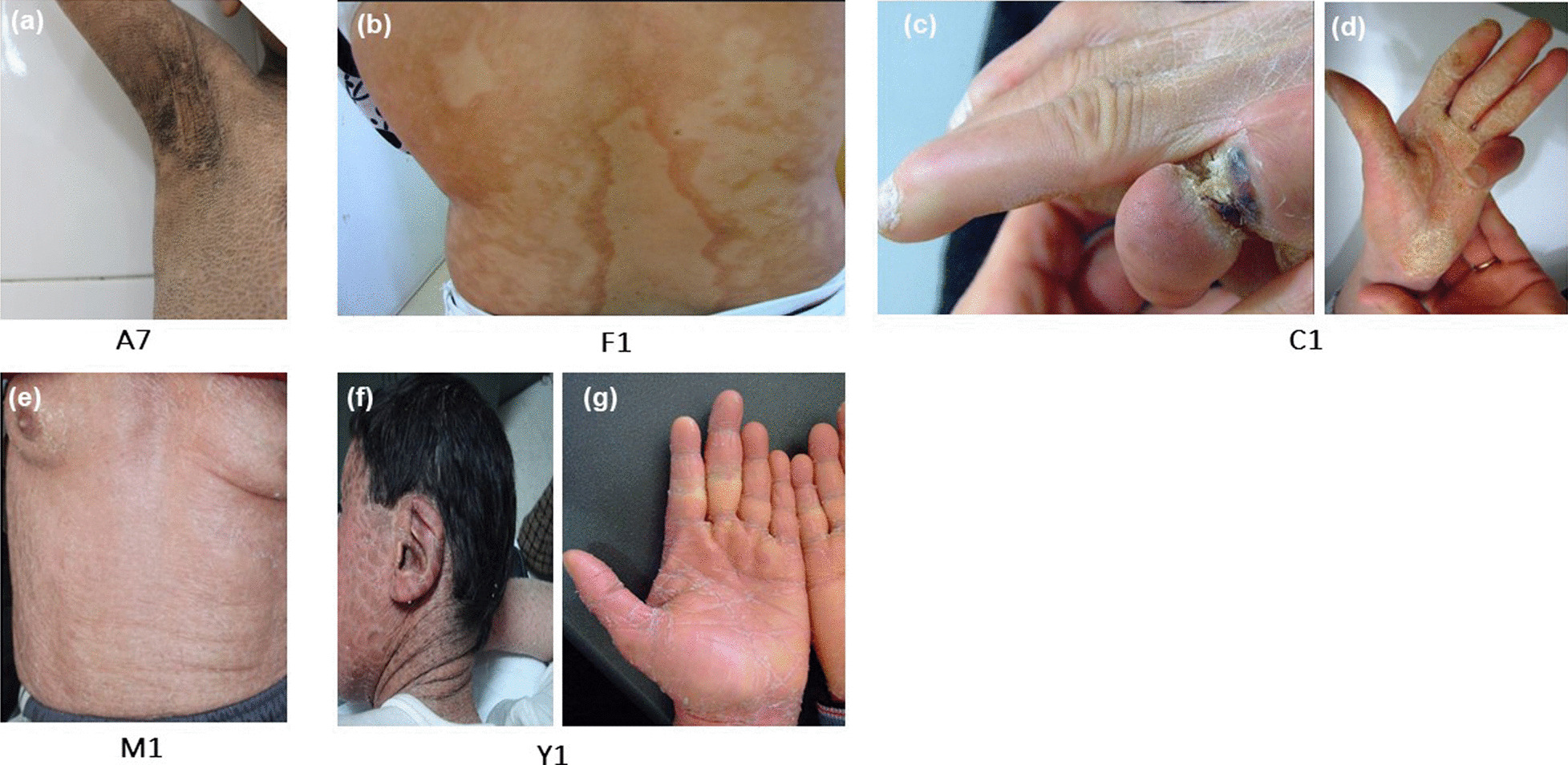
Clinical features of some studied patients. **a** Dark, thick and plate-like scales on the axilla of patient A7 (*NIPAL4*); **b** polycyclic erythematous and squamous plaques, with brownish fine scales on the trunk of patient F1 (*ABCA12*); **c** pseudoainhum of the right finger in patient C1 (*NIPAL4*), **d** a yellowish palmar keratoderma in patient C1 (*NIPAL4*); **e** skin of the trunk of patient M1 showing ichthyosiform erythroderma with fine, white scales (*TGM1*); **f** Patient Y1 displayed large brown plate-like scales on the face and neck with adhered ear (*TGM1*); **g** hands of patient Y1 showing diffuse palmar keratoderma and brachydactyly (*TGM1*)

All three patients from A family, were born as collodion babies. With age, A3 and A4 evolved to CIE, while A7 developed LI. At the age of one year, cutaneous examination of patient A4 showed generalized white fine scales with an erythematous underlying skin. Her brother (A3) displayed at the age of 3 years larger scales on the forehead and limbs, while the scales were fine and brown on the trunk on an erythematous skin. However, their first cousin (A7), who was affected with LI demonstrated darker, thicker and plate-like scales all over the body, without erythema (Fig. 1a). Patients with CIE (A3 and A4), showed sparse light brown hair compared to A7 and to the rest of the family members. Particularly, the folds were involved in A3 and A7, while hyperlinearity of the soles was noted only in A3. Patient A7 had a diffuse but not severe plantar keratoderma developed at the age of 6 years, while palms and soles were spared in patient A4.

The F family included 2 patients (F1 and F2). The proband F1, aged 35 years, had a similar phenotype to her 4 year-old daughter (F2). Both patients presented multiple polycyclic erythematous and squamous plaques, with brownish fine scales (Fig. 1b). Those lesions were located on the trunk in patient F1, and in the forearms, elbows and inguinal folds in the patient F2. This latter had cutaneous xerosis at birth. No ectropion, no alopecia and no ear malformation were noted in both patients.

C1 patient, who was 34 years old, had CIE with a mild erythema of the skin and small white scales sparing the folds. He presented a severe yellowish keratoderma of the palms and soles with a pseudo-ainhum and reduced fingers mobility (Fig. 1c, d). These aspects of extremities were very similar to those seen in Mal de Meleda disease. There was no ectropion and ears were normal.

M1 patient, aged of 74 years and also affected with CIE, showed mild erythroderma with fine white scales covering the whole body (Fig. 1e). He was born as a collodion baby and revealed, later, bilateral ectropion and diffuse palmoplantar keratoderma (PPK) or palmoplantar hyperlinearity. The folds were involved and alopecia was particularly noted. Brachydactyly with long palm and short fingers was also noted. Moreover, M1 patient suffered from painful palmar fissures and necrosis of the distal pulp of the second right finger and the third left finger secondary to repetitive infections.

On the other hand, B1, Y1 and K1 had LI phenotype. B1 patient aged one year, presented brown large plate like scales covering the entire body without erythema, while Y1 patient had black large plate like scales covering the entire body including the folds on an erythematous skin. Y1 displayed large brown plate-like scales on the face and neck with adhered ear (Fig. 1f). His hands showed diffuse palmar keratoderma (Fig. 1g). Both patients (B1 and Y1) were born as collodion babies and revealed, later, bilateral ectropion and diffuse PPK or palmoplantar hyperlinearity. The folds were involved and ears deformity was seen in both patients. Brachydactyly with long palm and short fingers was also noted. K1, aged 14 years, was born as a collodion baby and developed, later, large brown scales of the front, the trunk and proximal parts of the limbs. The scales became smaller on the legs and forearms. Large folds were spared. Mild palmoplantar keratoderma with hyperlinearity characterized the extremities. No ectropion, no nails abnormalities, no alopecia and no ears malformation were reported.

Finally, E1 patient affected with syndromic ichthyosis (CIE with ocular defect) presented mild erythematous skin. Scales were white and fine on the face, the trunk and the upper members, while they were thicker and larger on the knees and the legs. White scales were also located on the dorsal aspect of the hands and feet and in the folds. Mild palmoplantar keratoderma and brachydactyly were also observed. There was no ectropion and ears were normal. Interestingly, ophthalmological examination revealed bilateral myopia and microspherophakia.

### Molecular analysis

Using a multi-gene panel, we identified eight disease-causing variants in five different genes (*NIPAL4, TGM1*, *CYP4F22*, *ABCA12* and *CERS3*) (Table 3).

**Fig. 2:**
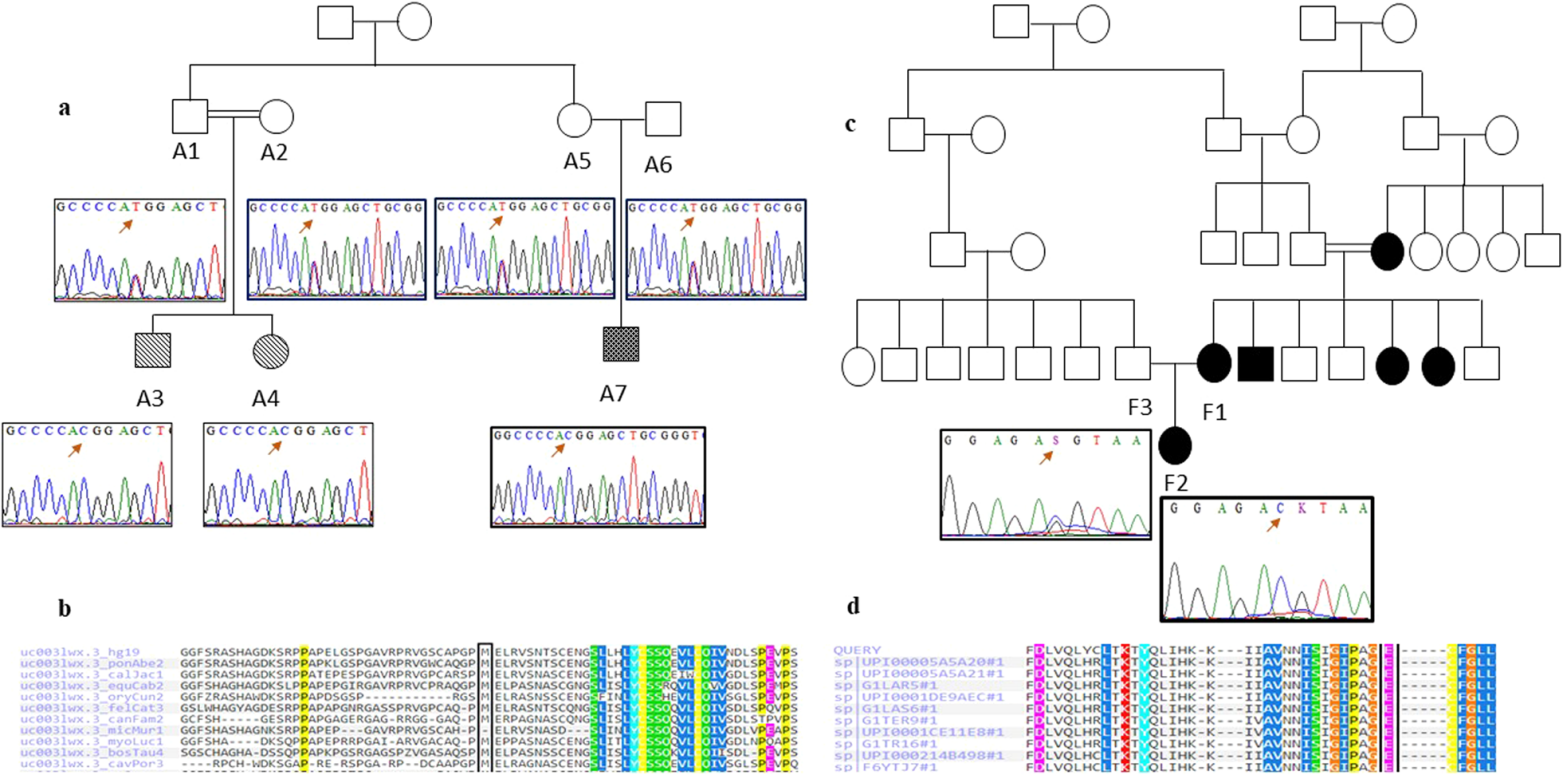
Example of sequence chromatograms and sequence alignments of novel missense mutations in *NIPAL4* and *ABCA12* genes in A and F families. **a** Sequence chromatograms of *NIPAL4*: c.188 T > C in A family at heterozygous and homozygous state in parents and affected children respectively; dotted shapes illustrate LI phenotype, hatched shapes illustrate CIE and black shapes illustrate ILC type **b** Sequence alignment of the NIPA4 protein in different species showing conservation of methionine residue throughout species is also given; **c** Sequence chromatograms of *ABCA12*: c.5898G > C in F family at homozygous and heterozygous state in father and affected children respectively **d** sequence alignment of the ABCA12 protein in different species showing conservation of Glu residue throughout species performed with polyphen

Mutational analysis of *NIPAL4* gene revealed two different germ line missense mutations in homozygous state in A family and C1 patient. The first one is a novel mutation (c.188T > C; ENST00000311946.7) in exon 1, which substitutes a methionine to a threonine (p.Met63Thr; ENSP00000311687.7). It was reported in family A affected members (A3, A4 and A7), with two ARCI phenotypes (CIE and LI). To confirm the segregation of this mutation and its association with ARCI, Sanger sequencing was performed in all available family members and control population. This mutation was in heterozygous state for parents and absent in unaffected members and control individuals (Fig. 2a).

The second disease-causing variant (c.534A > C; ENST00000311946.7) occurred in exon4 of *NIPAL4* gene and substituted glutamic to aspartic amino acid (p.Glu178Asp; ENSP00000311687.7). It was identified in a CIE patient (C1, 34 years old) who presented a mild erythema of the skin, with small white scales sparing the folds.

Two novel missense mutations were detected at the homozygous state: c.5898G > C (ENST00000389661.4) (p.Glu1966Asp; ENSP00000374312.4) and c.2855A > G (ENST00000389661.4) (p.Tyr952Cys; ENSP00000374312.4) in *ABCA12* gene in family F (F1 and F2 patients) with ILC (Fig. 2c). Segregation analysis confirmed the presence of these variations at the heterozygous state in the patient’s father. At the beginning, Netherton syndrome and Erythrokeratoderma variabilis phenotypes were suspected, but no hair shaft abnormalities were found and no mutations in *SPINK5* and *EKV* genes (*GJB2*, *GJB3*, *GJB4*, *GJB6* and *KDSR*) were identified (data not shown).

In three patients with non-syndromic ichthyosis (M1, B1 and Y1), the involved gene was *TGM1* with two different homozygous nonsense germline mutations (Table 3). Interestingly, both variations were described in gnomAD database in only heterozygous state. The most frequent mutation in this gene was c.788G > A (ENST00000206765.6) in exon 5 (p.Trp263X; ENSP00000206765.6) detected in M1 and B1 affected with CIE and LI respectively. The second recurrent disease-causing variant in *TGM1* gene was c.1042C > T (ENST00000206765.6) located in exon 7 (p.Arg348X; ENSP00000206765.6) and detected in patient Y1 aged 19 years, who showed LI phenotype.

K1 patient with LI had rather a single homozygous missense mutation (c.844C > T; ENST00000269703.3) in exon 8 which substitutes arginine to tryptophan (p.Arg282Trp; ENSP00000269703.1) in *CYP4F22* gene. This variation was also reported only in heterozygous state in gnomAD database.

We also identified a homozygous deletion in exon 13 of *CERS3* gene in a 15-year old patient (E1) affected with syndromic ichthyosis (CIE with ocular defect).

### Bioinformatic prediction

As mentioned in Table 3, and according to our in silico study, all novel missense variants were classified as damaging. Thus, no variant was expected to be a benign polymorphism using Polyphen2, SIFT, Mutation Taster, Panther and CADD together for the following mutations: p.Met63Thr in *NIPAL4* gene and p.Glu1966Asp, p.Tyr952Cys in *ABCA12* gene. We have to mention that all new variants were not reported before in the gnomAD database.

Moreover, multiple alignments of corresponding proteins showed that these residues were located within a highly conserved region (Fig. 2b, d).

## Discussion

We collected detailed phenotypic data from 11 Tunisian patients with different ichthyosis forms. In order to identify causative gene(s) mutation(s), we used a custom multi-gene panel. One of the most important aspects of this panel is its ability to be easily upgradable in view of novel discoveries. Moreover, compared with whole exome sequencing, analysis of the multigene panel is easier and faster. Overall, we identified 8 disease-causing variants in 5 different genes (*NIPAL4, TGM1, CYP4F22, ABCA12* and *CERS3*). Among these, three were novel (c.118C > T in *NIPAL4*; c.5898G > C and c.2855A > G in *ABCA12*). Using bioinformatic tools, these variations were predicted as pathogenic and causing a structural protein modification suggesting their involvement in ichthyosis. Moreover, already described mutations were pathogenic according to ClinVar database, except for c.844 C > T in *CYP4F22* gene which was classified as an uncertain significant variant. However, this variant was associated to both LI and CIE phenotypes [29, 30]. Moreover, c.845G > A (p.Arg282Gln) variant which is closed to c.844 C > T variation, was classified as likely pathogenic in ClinVar arguing the pathogenic effect of this mutation.

Unlike ARCI series in literature, where *TGM1* was the first responsible gene [31–33], in our cohort *NIPAL4* was rather the most involved gene (36%) with two missense mutations (c.534A > C and c.118T > C). This discordance could be explained by the small number of patients investigated in our study.

Phenotype-genotype correlation was one of the major objectives of the current investigation. Besides finding new variations in genes causing ichthyosis, we tried to highlight novel clinical characteristics associated with particular genotypes. In this context, a particular form of non-syndromic ichthyosis (ILC) with multiple localized polycyclic erythematous and squamous plaques and brownish fine scales was found in patients carrying two novel missense mutations in *ABCA12* gene. These variations and clinical related aspects have never been reported before. In fact, this gene is involved in either HI phenotype [34], with homozygous or compound heterozygous truncating mutations, or in CIE/LI with missense mutations [35–37]. On the other hand, ILC is quite different from Netherton syndrome which associates ILC with a specific hair-shaft abnormality and rather caused by mutations in *SPINK5* gene [38]. As clearly observed, no pathogenic variations in *SPINK5* gene, neither in genes involved in erythrokeratoderma variabilis (EKV) were found in ILC patients (data not shown), confirming then different etiologies. Recently, Leersum et al. [39] also reported a novel clinical phenotype of ichthyosis in a 3-year-old girl presenting a linear erythematosquamous lesions following the lines of Blaschko caused by *ABCA12* mosaicism. This particular form of ichthyosis was explained by the combination of a germline mutation and an acquired postzygotic mutation in this gene. *ABCA12* gene was reported also in a keratosis pilaris with missense mutation and in Nevus comedonicus with a high protein expression level in the sebaceous gland without any variation in *ABCA12* coding region [40].

Otherwise, our investigation showed that 75% of our patients with *NIPAL4* mutations presented with a phenotype of non-syndromic CIE. Concerning patients with *TGM1* mutations, 66.67% of them showed a phenotype of LI. These observations confirmed other studies showing that *NIPAL4* mutations were usually associated with a moderate phenotype with fine grey/white scales, while *TGM1* mutations were associated with a more severe phenotype with darker, adherent and plate-like scales [41].

Our findings showed that some clinical features were associated with particular genotypes (Table 4). Thus; we noted brachydactyly in all cases mutated in *TGM1* gene. This phenotype with ‘long palm/short fingers’ appearance was also reported in two Tunisian patients with LI carrying *TGM1* mutations [42]. Even though not reported in the literature, we suggest considering brachydactyly as indicative of *TGM1* mutations. To the best of our knowledge, this phenotype, noted in E1 patient also, has never been reported before in ARCI patients with *CERS3* mutations. We suggest that this symptom may be caused by the deletion of *ADAMTS17* gene [43]. Indeed, a founder homozygous contiguous gene deletion (including exon 13 of *CERS3*, complete sequence of non-coding RNA FLJ42289 and the first three exons of *ADAMTS17*) was reported in three consanguineous Tunisian families affected with ichthyosis associated with ocular, cardiac and skeletal anomalies [44]. Since E1 patient presented a similar skin phenotype with eye anomaly, and came from the same area, it was very likely that she carried the same described deletion [44]. In order to confirm this hypothesis, further analyses would be necessary.

**Table 4:** Clinical features and associated genes in studied ichthyosis patients

Gene	Clinical feature	Phenotype
*NIPAL4*	Collodion baby, Ectropion, Folds involvement, Palmoplantar hyperkeratosis	CIE, LI
*TGM1*	Brachydactyly, Collodion baby, Ectropion, Folds involvement, Palmoplantar hyperkeratosis, Ears malformation, Alopecia	CIE, LI
*CYP4F22*	Collodion baby, Palmoplantar hyperkeratosis	LI
*CERS3*(*ADAM17*)	Brachydactyly, Folds involvement, Palmoplantar hyperkeratosis	CIE^•^

*CIE* congenital ichthyosiform erythoderma; *LI* lamellar ichthyosis; *CIE*^•^ CIE with ocular defect

In our cohort, 7 patients were born as collodion babies and were mutated either in *TGM1* (3/7), *NIPAL4* (3/7), or *CYP4F22* (1/7) genes. In the literature, this clinical feature was significantly associated with *TGM1* [41], and *NIPAL4* mutations with varying frequencies (28–73%) [41, 45]. But it was also reported in patients carrying mutations in *ABCA12, CERS3, ALOX12B, ALOXE3, CYP4F22, lipase N, PNPLA1* and *SDR9C7* genes [46, 47].

Ectropion was noted in all studied patients with *TGM1* mutations, and 50% of those with *NIPAL4* mutations. This finding was consistent with literature data and confirmed the previous observation that ARCI patients bearing *TGM1* mutations were more likely to develop bilateral ectropion [47].

In our study, skin folds were spared in 2 patients with *NIPAL4* variation and in the patient with *CYP4F22* variation. Although reported in the literature [48] as a predilection site to be involved, large skin folds when spared could not rule out variations in *NIPAL4* and *CYP4F22* genes.

Our findings showed that palmoplantar keratoderma was associated with *NIPAL4, TGM1, CYP4F22* mutations and *CERS3* deletion, but with varying severity. Thus, in patients with p.Met63Thr *NIPAL4* variation PPK was either moderate or absent. However, PPK was rather yellowish and severe in patients with p.Glu178 Asp *NIPAL4* mutation as reported in the literature. Indeed, yellowish PPK has been reported in *NIPAL4* series and, to the best of our knowledge, never associated with other ARCI genes [45]. Thus, yellowish keratoderma could be indicative of *NIPAL4* mutation in ARCI patients. In addition, patient C1 showed a constricting band around his right fifth finger, suggestive of pseudoainhum (Fig. 1b). The latter is a rare complication of ARCI and several other disorders of keratinization such as Vohwinkel syndrome and Mal de Meleda [49]. It has been suggested that pseudoainhum may be the consequence of repetitive infections or constriction by a thick plate of keratin [49]. However, the exact pathogenic mechanism in ARCI remains unclear.

In our *TGM1* mutated patients, we reported mainly a hyperlinearity of the palms and soles, with sometimes a severe PPK.

Ears deformity was reported in two patients (B1 andY1) and alopecia in one patient (M1) carrying *TGM1* mutation. Ears deformity was more commonly found in *TGM1*, *ABCA12*, *ALOX12B* and *ALOXE3* mutations compared to other ARCI forms [41]. Alopecia was found to be significantly associated with *TGM1* mutations.

Nevertheless, we also revealed that diverse phenotypes were caused by the same gene with an identical mutation. Notably, c.788G > A in *TGM1* gene was found in both CIE (M1 patient) and LI (B1 patient), suggesting the effect of a modifier gene as it was already reported [50]. In an attempt to explain this phenotypic heterogeneity, we examined *FLG* gene coding the filaggrin protein. We identified 44 missense polymorphisms in M1 not present in B1 (data not shown). Prediction effect of these polymorphisms showed that the majority were benign.

To summarize, we report three novel mutations, two of which were located in *ABCA12* gene responsible for ILC, a rare clinical form of ichthyosis, as well as novel clinical characteristics associated with particular genotypes.

## Conclusions

Phenotypic-genotypic correlation suggests that brachydactyly could be related to *TGM1* mutations and associated to deletion of *ADAMTS17* gene associated to deletion of *CERS3* exon 13.

Besides the new reported variations in *NIPAL4* and *ABCA12* genes, we added new insights to the already reported particular phenotypes linked to specific genes. The involvement of such genes in a particular ARCI form remains discussed. In order to better explain its phenotype heterogeneity, investigation of whole genome is necessary to search for responsible modifier genes.

### Web resources

http://bioinfo.ut.ee/primer3-0.4.0/

http://www.ncbi.nlm.nih.gov/blast/

https://www.genome.jp/tools-bin/clustalw

http://genetics.bwh.harvard.edu/pph2/

https://sift.bii.a-star.edu.sg/

http://www.mutationtaster.org/

http://www.pantherdb.org/tools/index.jsp

https://cadd.gs.washington.edu/snv

## Data Availability

The datasets used and/or analyzed during the current study are available in NCBI SRA at https://dataview.ncbi.nlm.nih.gov/object/PRJNA765516?reviewer=pu6eruli4285rvodofg5o2qndc.

## Abbreviations

ARCI: Autosomal recessive congenital ichthyosis
ILC: Ichthyosis linearis circumflexa
CIE: Congenital ichthyosiform erythroderma
LI: Lamellar ichthyosis
HI: Harlequin ichthyosis
NGS: Next generation sequencing
